# Comment on “Biosimilar FSH preparations- are they identical twins or just siblings?”

**DOI:** 10.1186/s12958-016-0196-3

**Published:** 2016-09-26

**Authors:** Thomas Strowitzki, Waldemar Kuczynski, Arnd Mueller, Peter Bias

**Affiliations:** 1 Department of Gynecological Endocrinology and Reproductive Medicine, University of Heidelberg, Im Neuenheimer Feld 440, 69120 Heidelberg, Germany; 2 Centre for Reproductive Medicine, Cryobank, 15879 Bialystok, Poland; 3 Department of Gynaecology and Gynaecological Oncology, Medical University, 15089 Bialystok, Poland; 4 Merckle GmbH, a member of the Teva group, 89079 Ulm, Germany

**Keywords:** Follitropin alfa, Recombinant human follicle-stimulating hormone (r-hFSH), Assisted reproductive technologies (ART), Biosimilar, Ovaleap, Gonal-f, Bemfola, Infertility

We read with interest the editorial by Orvieto and Seifer [1] on the EU approved r-hFSH biosimilar products Ovaleap® [2] and Bemfola® [3] and the reference Gonal-f®. We were surprised by the negative tone and seeming dismissal of EMA guidelines [4] for the development and marketing authorization of r-hFSH biosimiliars. The editorial questions the legal basis for r-hFSH biosimilar registration and use in clinical practice. We comment on the development and role of r-hFSH biosimilars and correct errors and omissions in the editorial.

The phase 3 Bemfola® and Ovaleap® populations are referred to as “ideal” patients in the editorial. Not recognized is that the phase 3 studies that supported Gonal-f® and Puregon® used similar patient populations and additional data were generated after the products became available for clinical use.

After acknowledging that the mean number of oocytes retrieved (EMA recommended primary efficacy outcome) was equivalent with the reference Gonal-f® and safety was comparable, the editorial re-interprets the non-significant differences between groups in pregnancy and OHSS (secondary endpoints) and suggests the biosimilars and reference r-hFSH products are not comparable. The editorial pooled Bemfola® and Ovaleap® data but did not include the caveat that different study protocols were used (e.g., randomization schemes, different GnRH agonists, dose adaptations). These differences likely place limitations on pooled comparisons. Importantly, number of oocytes retrieved is a clinically meaningful surrogate for successful live birth [5], the primary aim of IVF treatment. Pregnancy and live birth rates are problematic as they may be influenced by factors beyond r-hFSH treatment – date of embryo transfer, embryo transfer regional policies, luteal phase support, etc…

Clinically meaningful errors or omissions were made that favored the superiority of Gonal-f® over the biosimilar product:

- “Gonal-f® group achieved non-significantly more oocytes” than the Bemfola® group. However, this refers to FSH treatment duration and not to the number of oocytes retrieved. Interestingly, it was the Bemfola® group that achieved non-significantly more oocytes.
- “the Gonal-f® group achieved non-significantly lower peak E2 levels and lower cancellation rate with the consequent decreased incidence of OHSS.” However, study data [2] showed that cancellation rate was actually lower in the Ovaleap® group. And, this statement suggests a link between “non-significantly lower” E2 peak level and “decreased incidence” of OHSS, even though OHSS rates were not significantly different between groups (*P* = 0.542) [2]. Although OHSS incidence was low in both groups, it led to more treatment discontinuation with Gonal-f® (2) than with Ovaleap® (1).

## Conclusion

The editorial appears to argue that biosimilar products should not be considered in clinical practice, constraining the role of r-hFSH biosimilars as simply “a *regulatory synonym*," thereby promoting the reference product as the sole treatment option. We maintain women should have access to approved alternative treatment options with demonstrated comparable efficacy and safety, as occurs in the development of r-hFSH biosimilar products in response to EMA guidelines. Such options may improve health care and lower financial burden. Clinicians should be able to select an approved r-hFSH product at their discretion based on individual patient characteristics and clinical needs.

## Author response

### Reply to comment on “Biosimilar FSH preparations- are they identical twins or just siblings?”

^1^Raoul Orvieto MD, MMSc and ^2^David B. Seifer MD

^1^Infertility and IVF Unit, Department of Obstetrics and Gynecology, Chaim Sheba Medical Center (Tel Hashomer), Ramat Gan, Israel and Sackler Faculty of Medicine, Tel Aviv University, Tel Aviv, Israel.

^2^Division of Reproductive Endocrinology and Infertility, Department of Obstetrics and Gynecology, Dartmouth-Hitchcock Medical Center, Geisel School of Medicine at Dartmouth, Lebanon, NH, USA

We thank Strowitzki et al. for their letter. The editorial was not meant to be negative to any product (biosimilar or the reference products), or to recommend against the use of biosimilar products in clinical practice, but to describe the biological differences between the products and to provide a brief overview of the published clinical studies.

Biosimilars are legitimate products, and are a welcomed addition to the FSH armamentarium. We have not “questioned the legal basis” of biosimilar registration, but rather, have called for caution while implementing a new product to the COH armamentarium. As we have already stated and clearly described, since biosimilars are not identical to the reference products, and due to the fact that the clinical experience gained with their use came from RCT’s which included “ideal”, best prognosis patients, we believe that *for patients’ safety*, “further comparative studies are needed in other patient populations that are encountered during routine daily clinical practice, e.g., older, poor responders, patients with repeated IVF failures or high responders, such as those with polycystic ovary syndrome, before the *universal* implementation of biosimilar products to clinical use”. Moreover, this is also why we recommend “against interchanging or substituting innovator and biosimilar agents in clinical practice, and believe that the decision whether to use an innovator or a biosimilar product, should be reserved to the discretion of the treating physician”.

Specific comments:

We do not doubt, neither did we attempt to challenge the legitimacy of the biosimilar products registration, the EMA regulations or decisions. As mentioned above, biosimilars are legitimate products and are a welcomed addition to the FSH armamentarium.

The fact the RCTs (those supporting the use of Gonal-f and Puregon) were conducted in “ideal” patients, does not mean that caution should not be taken while treating “non-ideal” patients.

The clinical data, as presented in the Editorial, summarized the two RCT’s and did not mean to replace them. As stated, Table 2 simply gathered the data from the 2 RCTs and was not meant to conduct a sophisticated statistical analysis. Its intent was to support the notion that biosimilars are not “identical twins”. Moreover, the cancellation rate was non-significantly lower in the Gonal-f, as compared to the Bemfola group. No mention was made in the Editorial regarding cancellation rate in the Ovaleap Study.

We thank Strowitzki et al. for their correction regarding the oocyte number (which was stated correctly in the table, but not in the text). An erratum has been published accordingly.

## Abbreviations

ART: assisted reproductive technologies
EMA: European Medicines Agency
FDA: US Food and Drug Administration
GnRH: gonadotropin-releasing hormone
IVF: in vitro fertilization
OHSS: ovarian hyperstimulation syndrome
r-hFSH: recombinant human follicle-stimulating hormone
